# Influence of e-Beam Irradiation on the Physicochemical Properties of Poly(polyol Succinate-co-Butylene Succinate) Ester Elastomers

**DOI:** 10.3390/ma13143196

**Published:** 2020-07-17

**Authors:** Marta Piątek-Hnat, Kuba Bomba, Jakub Pęksiński, Agnieszka Kozłowska, Jacek G. Sośnicki, Tomasz J. Idzik, Danuta Piwowarska, Jolanta Janik

**Affiliations:** 1 Department of Polymer and Biomaterials Science, Faculty of Chemical Technology and Engineering, West Pomeranian University of Technology, Piastów Ave. 42, 71-065 Szczecin, Poland; bk34688@zut.edu.pl (K.B.); agak@zut.edu.pl (A.K.); jolanta.janik@zut.edu.pl (J.J.); 2 Faculty of Electrical Engineering, West Pomeranian University of Technology, Sikorskiego Ave. 37, 71-313 Szczecin, Poland; jakub.peksinski@zut.edu.pl; 3 Department of Organic and Physical Chemistry, Faculty of Chemical Technology and Engineering, West Pomeranian University of Technology, Piastów Ave. 42, 71-065 Szczecin, Poland; jacek.sosnicki@zut.edu.pl (J.G.S.); tomasz.idzik@zut.edu.pl (T.J.I.); 4 Department of Technical Physics, Faculty of Mechanical Engineering and Mechatronics, West Pomeranian University of Technology, Al. Piastów 48, 70-310 Szczecin, Poland; danuta.piwowarska@zut.edu.pl

**Keywords:** e-beam modification, mechanical and thermal properties, sugar alcohols, ester elastomers

## Abstract

The purpose of this research was synthesis and electron beam modification of novel ester elastomers consisting of sugar alcohol–succinic acid block and butylene glycol–succinic acid block. Four different alditols were used in the synthesis—sorbitol, erythritol, xylitol, and glycerol. The materials were irradiated with doses of 50, 100, and 150 kGy in order to determine which dose is the most beneficial. As expected, irradiation of the materials has led to the cross-link density becoming higher and improvement of the mechanical properties. Additionally, the materials were also sterilized in the process. The great advantage of elastomers described in the paper is the fact that they do not need chemical cross-linking agents or sensitizers in order to undergo radiation modification. The following tests were performed on cross-linked poly(polyol succinate-co-butylene succinate) elastomers: quasi-static tensile test, determination of cross-link density, differential scanning calorimetry (DSC), dynamic thermomechanical analysis (DMTA), wettability (water contact angle), and Fourier transform infrared spectroscopy (FTIR). In order to confirm successful synthesis, prepolymers were analyzed by nuclear magnetic resonance spectroscopy (^1^H NMR and ^13^C NMR).

## 1. Introduction

Increasing demand for engineering plastics with very good mechanical properties has made it necessary to find a way to greatly enhance properties of common polymers in a cost-efficient manner that allowed mass production. Radiation modification is such a method. In addition to being economical in terms of saving energy, space, and time, it also allows for a high degree of control over the cross-linking process and eliminates the risk of microvoids being present in the material. In addition to mechanical properties, chemical and thermal characteristics are also improved in the process. Radiation modification has many commercial applications, such as cross-linking polyethylene (PE), polypropylene (PP), and poly(vinyl chloride) (PVC) for wire insulation, curing rubber compounds for tires, production of cross-linked PE pipes, and heat-shrinkable polyolefine tubing [1,2].

In case of polymers with possible medical uses, radiation modification serves a double role—in addition to improving the properties, it also sterilizes the material. Most researched polyesters belonging to this group are polycaprolactone (PCL) and polylactide (PLA). Radiation modification of PCL with chemical cross-linkers, including polyester acrylate [3], triallyl isocyanurate (TAIC) [4], and without any cross-linking agents [5], was reported. PLA was successfully radiation cross-linked both without any cross-linking agents [6] and with addition of TAIC [7,8,9]. 

Radiation modification of other polyesters was also reported, such as poly(butylene succinate) (PBS) with addition of trimethallyl isocyanurate (TMAIC) [10], various thermoplastic elastomers with and without cross-linking agents [11,12], poly(ethylene terephthalate) impregnated with radiation sensitizer—trimethylopropane triacrylate (TMPTA) [13,14], poly(butylene adipate-co-terephthalate) (PBAT) with TAIC [15], poly(butylene terephthalate) (PBT) with triallyl cyanurate [16], and without any cross-linking agents [17]. Bacterial polyesters were also radiation cross-linked without requiring any cross-linking agents [18,19].

In case of polyesters based on sugar alcohols, as far as we know, the only reported use of radiation by other authors was conducted in very low doses in order to only sterilize the material and not modify its properties [20]. 

In general, materials synthesized utilizing sugar alcohols as monomers have desirable properties, making them a perfect candidate for further research. They are not only biodegradable, but also biocompatible [21,22]. While retaining this core characteristic, their properties can be fine-tuned by changing the monomer chain length and monomer ratio [23], by using different sugar alcohols [24,25], or by introducing a diol to the synthesis [26,27,28]. In our previous work, we have synthesized and tested various sugar alcohol-based polyesters [29,30,31]. We have also used radiation to modify different poly(polyol succinate-co-butylene succinate) elastomers [32]. Based on our previous paper, we have deemed it scientifically valuable to radiation-modify a group of sugar alcohol-based polyester based on a dicarboxylic acid with a much shorter chain length—succinic acid. It is of note that our polyesters do not require addition of chemical cross-linking agents or sensitizers. It is very important since such substances could leave potentially harmful residuals in the material after cross-linking, which makes them much less desirable in potential food packaging or medical uses.

## 2. Materials and Methods

### 2.1. Synthesis of Elastomers and Sample Preparation

Four alditols (erythritol, sorbitol, glycerol, and xylitol), succinic acid, and butanediol (Sigma-Aldrich, St. Louis, MO, USA) were used to synthesize four polymers—poly(glycerol succinate-co-butylene succinate) (PGBSu), poly(erythritol succinate-co-butylene succinate) (PEBSu), poly(xylitol succinate-co-butylene succinate) (PXBSu), and poly(sorbitol succinate-co-butylene succinate) (PSBSu).

The succinic acid:alditol:butylene glycol monomer ratio used was 2:1:1. Synthesis was carried out according to the procedure described in previous papers [29,30,31,32]. Esterification of succinic acid, alditol, and butylene glycol for 13.5 h in 150 °C and N_2_ atmosphere was the first step of the synthesis. Next, 3.5 h of polycondensation reaction in vacuum in 150 °C took place. The materials obtained directly after polycondensation are called “prepolymers” further in the text. Following polycondensation, prepolymers were cast into forms made of silicon. After that, cross-linking in a vacuum dryer in 100 °C and 100 mBar atmosphere was carried out. Samples for further tests were cut from the cross-linked sheets of the material using a punching die.

### 2.2. Irradiation

E-beam irradiation was performed on the cross-linked samples. The procedure was carried out in the Institute of Nuclear Chemistry and Technology (Warsaw, Poland). Elektronika 10/10 (NPO Torij, Moscow, Russia) linear electron accelerator was utilized. The parameters of the process were as follows: 360 mA average set current, 10 MeV beam, and 0.368 m/min sample moving speed. Standard [33] was adhered to, and 50, 100, and 150 kGy radiation doses were used.

### 2.3. Experimental Methods

#### 2.3.1. Nuclear Magnetic Resonance Spectroscopy (NMR) 

Bruker DPX 400 AVANCE III HD spectrometer (Bruker, Rheinstetten, Germany) was used to carry out the ^13^C and ^1^H NMR analyses. The parameters of the process were as follows: 400.1 MHz for ^1^H analysis and 100.6 mHz for ^13^C analysis. Five-millimeter probes were used to obtain the spectra. Deuterated chloroform (CDCl_3_) was used as solvent. The weight of each sample was approximately 50 mg, and the volume of the solvent was 0.7 mL. The internal reference used was tetramethylsilan (TMS). Development of the results was done with the following software: MestReNova 12.03 (Mestrelab, Santiago de Compostela, Spain).

#### 2.3.2. Fourier Transform Infrared Spectroscopy (FTIR)

Bruker Alpha Spectrometer (Bruker, Rheinstetten, Germany) was utilized to perform Fourier transform infrared spectroscopy (Attenuated Total Reflection (ATR) FTIR). The parameters of the procedure were as follows: 4000 to 400 cm^−1^ range, 2 cm^−1^ resolution, 32 scans. Development of the results was done with following software: Omnic 7.3 (Thermo Electron Corporation, Waltham, MA, USA). Both non-irradiated and radiation-modified materials were analyzed. 

#### 2.3.3. Differential Scanning Calorimetry (DSC)

Q2500 DSC instrument (TA instruments, New Castle, DE, USA) was utilized to perform differential scanning calorimetry. The following parameters were used: nitrogen atmosphere, heating rate of 10 °C/min, heating cycle from −100 to 100 °C. Both non-irradiated and radiation-modified materials were analyzed. Development of the results was done with the following software: 3.9a TA Instruments Universal Analysis 2000 (New Castle, DE, USA).

#### 2.3.4. Dynamic Thermomechanical Analysis (DMTA)

DMA Q800 (TA Instruments, New Castle, DE, USA) was utilized to carry out dynamic thermomechanical analysis (DMTA). The following parameters were used: −100 to 100 °C temperature range, heating rate of 2 °C/min, 1 Hz frequency. Development of the results was done with the following software: 3.9a TA Instruments Universal Analysis 2000 (New Castle, DE, USA). Both non-irradiated and radiation-modified materials were analyzed.

#### 2.3.5. Mechanical Properties

Instron 36 (Norwood, MA, USA) was utilized to carry out the tensile tests. The following parameters were used: relative humidity of 50%, 100 mm/min speed of the crosshead, 25 °C, 500N load cell. Standard [34] was adhered to. Both non-irradiated and radiation-modified materials were tested.

#### 2.3.6. Water Contact Angle

Digital goniometer KRUSS DSA100 (Hamburg, Germany) was utilized to carry out the water contact angle test. An automatic dispenser was utilized to place 2 µL of deionized water on the material surface, which was previously degreased. Development of the results was done with the following software: DSA4 drop shape analysis (Kruss, Hamburg, Germany). Both non-irradiated and radiation-modified materials were tested.

#### 2.3.7. Cross-Linking Density

Three samples (each weighing about 2 g) of all materials, both before and after irradiation, were prepared. Tetrahydrofuran (THF) was used to submerge every sample. The volume of THF used for each sample was 20 mL. The temperature was 20 °C, and the time of immersion was 5 days. Next, separation of each sample from the solvent was carried out, and the samples were weighed in order to measure the wet fraction. After that, 8 days of drying in 20 °C in a vacuum dryer was carried out. The dried samples were weighed in order to measure the dry fraction.

Calculation of cross-link density was done using the Flory-Rehner Equation [35]

(1) $$v=\frac{\ln\left(1-v_{2}\right)+v_{2}+\chi v_{2}^{2}}{v_{1}\left(\left(\frac{v_{2}}{2}\right)-v_{2}^{\frac{1}{3}}\right)}$$

(2) $$v_{2}={\left[1+\left(\frac{m_{1}-m_{2}}{m_{2}}\right)\left(\frac{\rho_{s}}{\rho_{\rho }}\right)\right]}^{-1}$$

The meaning of the symbol is as follows: *v*—½ of the number of active network chain segments per unit volume (cross-link density, *n*), χ—polymer–solvent interaction parameter (*χ* = 0.42, as determined for sugar alcohol-based materials [36]), *v*_2_—polymer volume fraction at equilibrium swelling, $\rho_{p}$—polymer density, $\rho_{s\;}$—solvent density, *v*_2_—the polymer volume fraction, *v*_1_—solvent molar volume at equilibrium swelling, *m*_1_—wet fraction weight, and *m*_2_—dry fraction weight.

#### 2.3.8. Hardness

Hardness (H) for materials before and after irradiation was measured using a Zwick/Material Testing 3100 Shore A hardness tester (ZwickRoell, Kennesaw, GA, USA)

#### 2.3.9. Gel Permeation Chromatography

Gel Permeation Chromatography (GPC) was utilized in order to determine the molecular weights of PGBSu, PEBSu, PXBSu, and PSBSu. Tests were conducted using Styragel column (Waters, Milford, CT, USA), with tetrahydrofuran (THF) as solvent in which samples (1 mg/mL) were dissolved.

## 3. Results and Discussion

**Figure 1:**
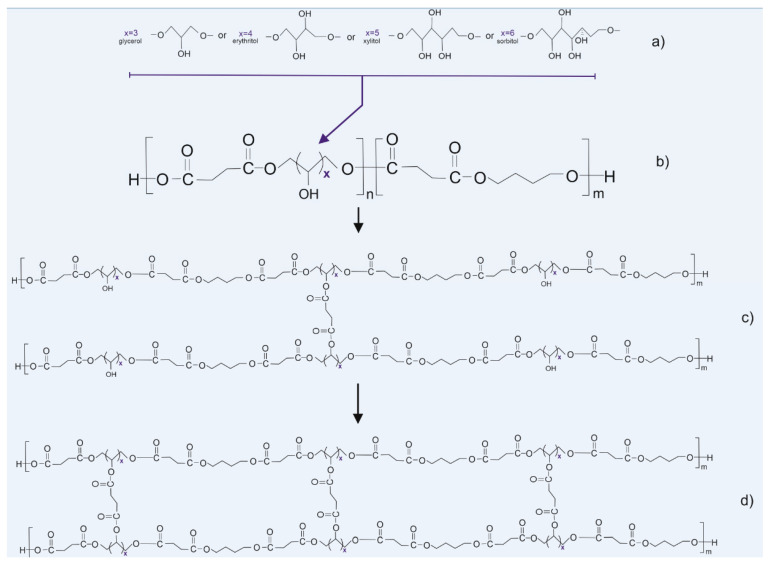
Scheme of synthesis and modification of the polymers: (**a**) sugar alcohols used in the synthesis, (**b**) prepolymer structure, (**c**) thermally cross-linked polymer, and (**d**) radiation cross-linked polymer.

Table 1 and Table 2 contain the polymer composition and properties; Figure 1 presents the polymer structure scheme and suspected scheme of radiation-induced crosslinking.

### 3.1. Nuclear Magnetic Resonance Spectroscopy (NMR)

The success of the synthesis was confirmed, and the polymer structure was analyzed by ^13^C NMR and ^1^H NMR. 

**Figure 2:**
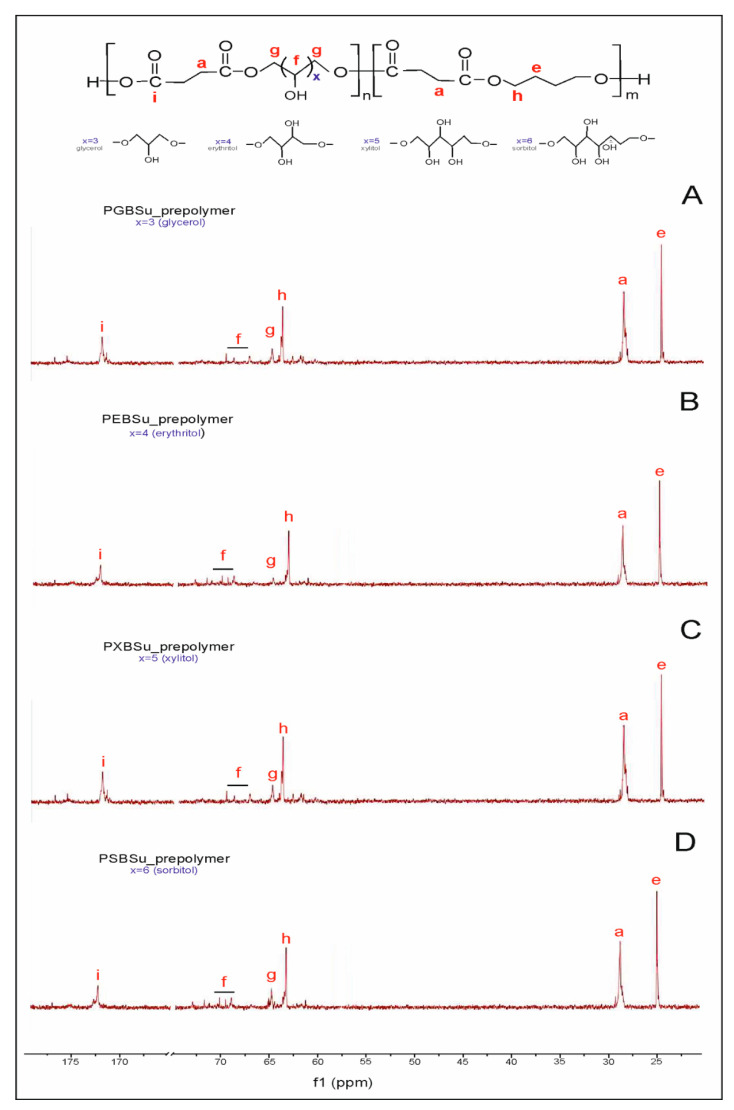
^13^C NMR of PGBSu (**A**), PEBSu (**B**), PXBSu (**C**), and PSBSu (**D**) prepolymers.

In ^13^C NMR (Figure 2), two peaks linked to the CH_2_ groups can be seen—peak at 25 ppm corresponding to the CH_2_(e) groups and peak at 30 ppm connected to the CH_2_(a) group. Two peaks at approximately 65 ppm are present—the first one was ascribed to a carbon atom C(h) adjacent to an ester bond between dicarboxylic acid and butylene glycol, and the second one was attributed to a carbon atom C(g) next to an ester bond between dicarboxylic acid and sugar alcohol. The peak at approximately 170 ppm is linked to a carbon atom C(i) in a carbonyl group in dicarboxylic acid.

**Figure 3:**
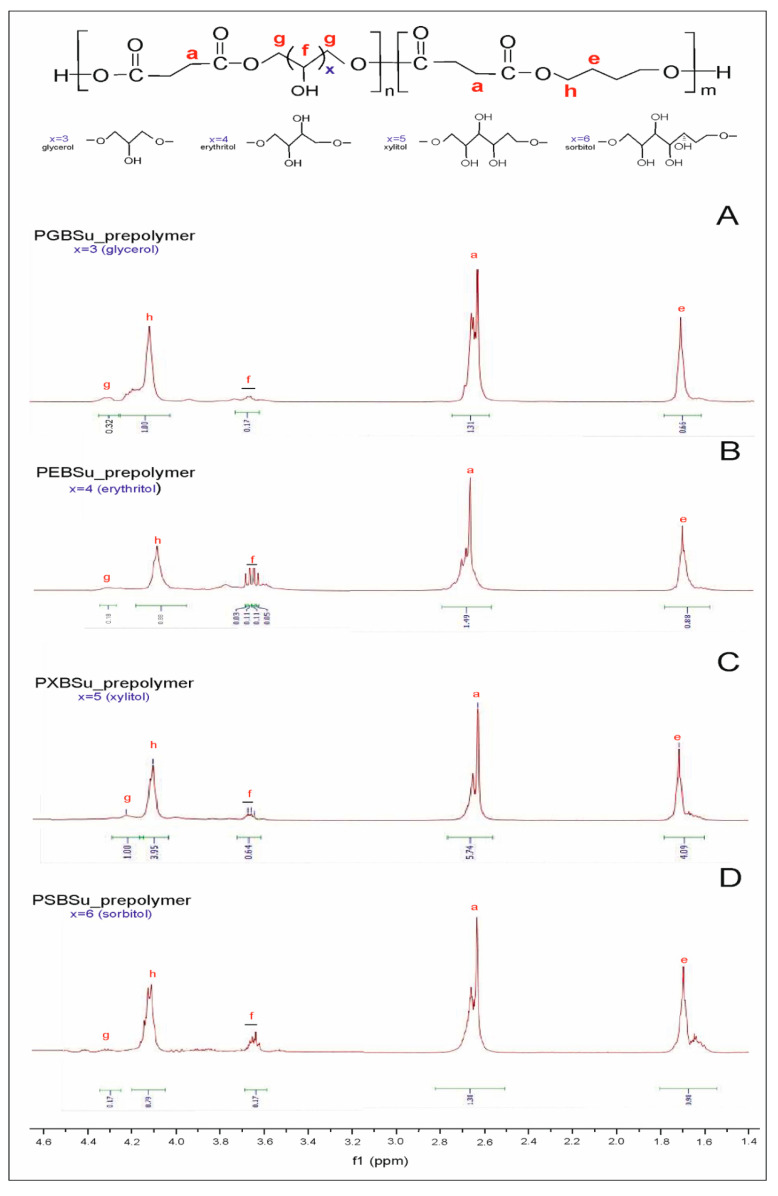
^1^H NMR of PGBSu (**A**), PEBSu (**B**), PXBSu (**C**), and PSBSu(**D**) prepolymers.

In ^1^H NMR (Figure 3), two peaks connected to the CH_2_ groups are observed—the signal at 1.7 ppm linked to the CH_2_(e) groups, and the signal at 2.7 ppm corresponding to the CH_2_(a) group. Two peaks linked to hydrogen atoms adjacent to ester bonds are present—one at 4.1 ppm linked to the hydrogen atom (h) in butylene glycol and one at 4.3 ppm connected to the hydrogen atom (g) in sugar alcohol. The peaks in the range of 3.6–3.8 ppm were connected to the CH_2_ (f) groups in sugar alcohol. By comparing the signal integration of peaks linked to the CH_2_(h) and CH_2_(g) groups, we have calculated the molar composition of the polymers. The difference between the molar composition in the feed and the actual molar composition calculated by ^1^H NMR is due to the alditols having lower reactivity as compared with butylene glycol. This reactivity also varies between different sugar alcohols.

### 3.2. Fourier Transform Infrared Spectroscopy (FTIR)

**Figure 4:**
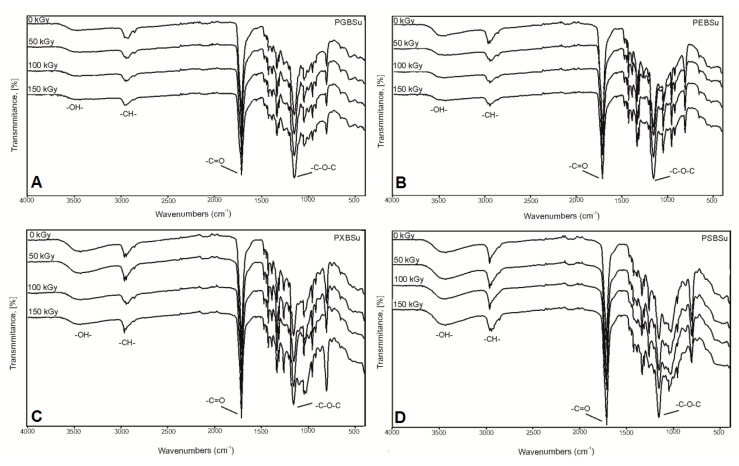
FTIR spectra of PGBSu (**A**), PEBSu (**B**), PXBSu (**C**), and PSBSu (**D**) polymers before and after radiation treatment.

In the FTIR (Figure 4), four peaks linked to groups typical for poly(polyol succinate-co-butylene succinate) elastomers are present: -OH groups at 3450 cm^−1^, -CH groups at 2930 cm^−1^, C=O groups at 1725 cm^−1^, and –C–O–C groups at 1170 cm^−1^, which confirms a successful synthesis. A small change in the intensity of signals connected to the hydroxyl groups was observed when comparing the spectra for the material before and after irradiation—it decreases for PEBSu and PXBSu and increases for PSBSu. It is connected to the cross-link density results—the cross-link density increases for PEBSu and PXBSu and decreases for PSBSu. It is caused by ester bond formation between chains in case of all materials except for PSBSu, in case of which, those bonds undergo scission. 

Lack of significant changes in the spectra of materials after modification confirms that the ester structure is retained after applying radiation treatment. 

### 3.3. Thermal Properties: Differential Scanning Calorimetry (DSC)

**Figure 5:**
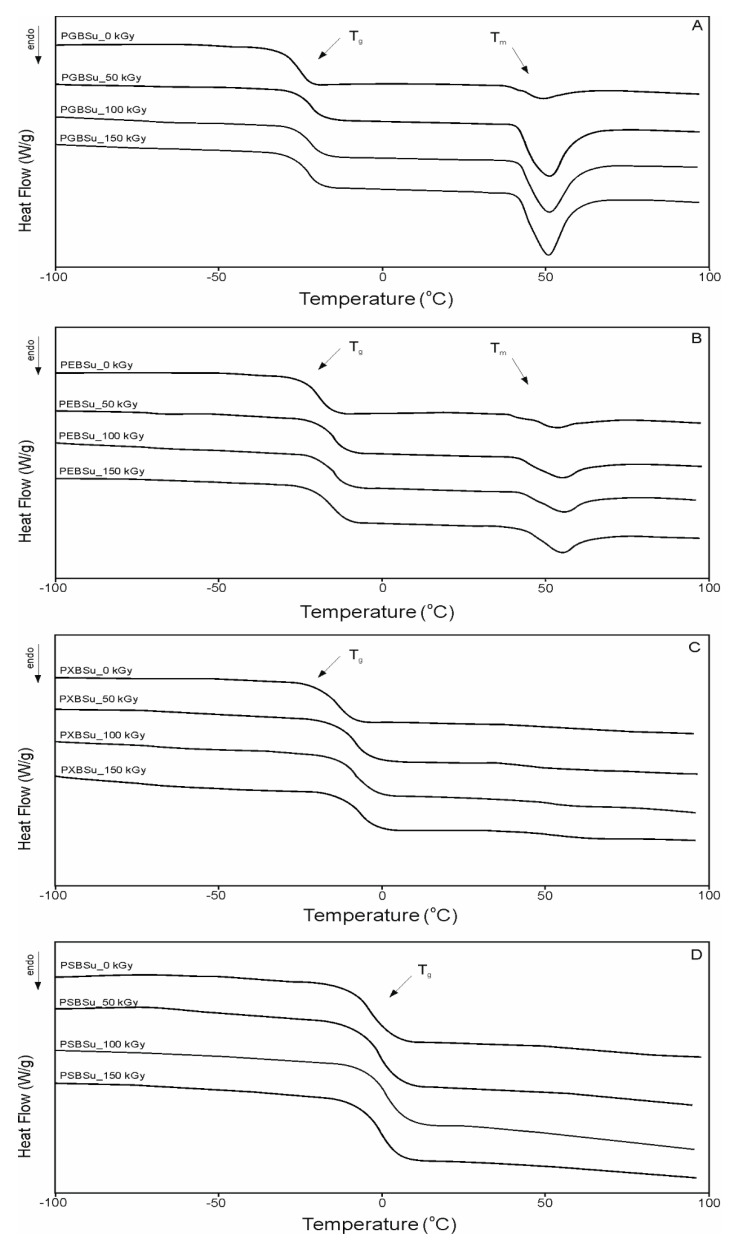
Differential scanning calorimetry (DSC) thermograms for first heating. (**A**) PGBSu, (**B**) PEBSu, (**C**) PXBSu, and (**D**) PSBSu before and after irradiation.

DSC analysis (Table 3 and Figure 5) was conducted in order to see how radiation modification affects the thermal properties of poly(polyol succinate-co-butylene succinate) elastomers. The value of change in heat capacity does not differ much between polymers based on various sugar alcohols and between different radiation doses due to the amount of amorphous phase being at a similar level in all cases. However, glass transition temperature shows an upward tendency linked to increase in the amount of –OH groups, which is linked to a lower chain mobility and corresponds to a higher value in cross-link density as shown in Section 3.5 of this paper. Glass transition temperature also increases due to radiation treatment and further cross-linking taking place, and corresponds with higher cross-link density. 

Values of the melting enthalpy show a downward tendency corresponding with the increase of the amount of –OH groups and the increase of cross-link density (for materials before modification). Radiation treatment leads to the melting temperature and enthalpy due to crystalline structure becoming more ordered.

### 3.4. Dynamic Thermomechanical Analysis (DMTA)

**Figure 6:**
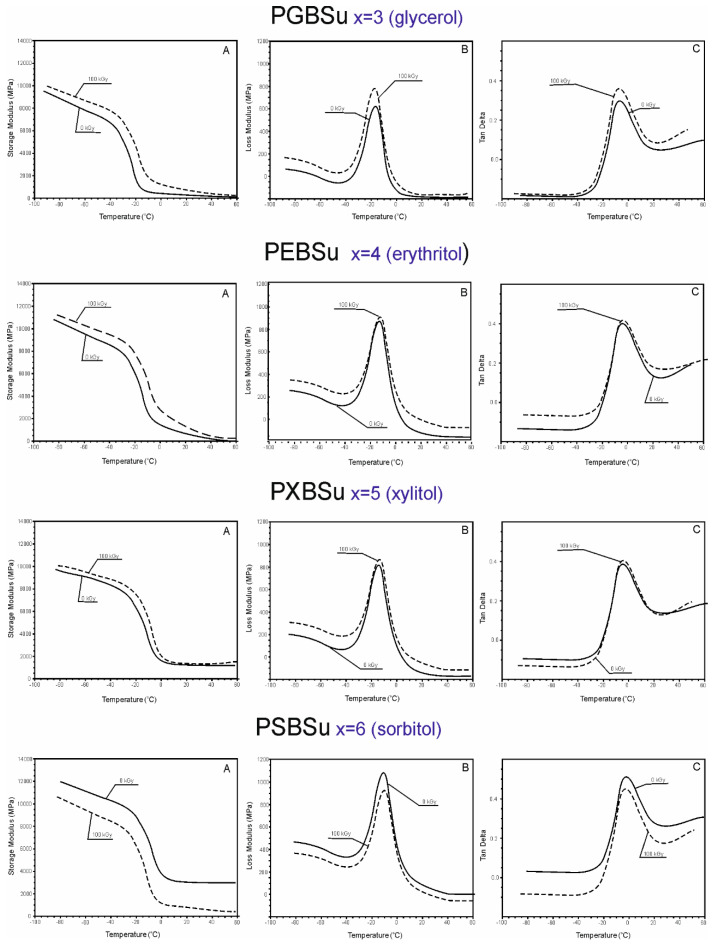
Dynamic thermomechanical analysis (DMTA). (**A**) Storage modulus (E’), (**B**) loss modulus (E”), and (**C**) loss tangent (tan delta versus temperature) for PGBSu, PEBSu, PXBSu, and PSBSu.

DMTA (Figure 6) was used to test the relaxation behavior displayed by PGBSu, PEBSu, PXBSu, and PSBSu (before and after radiation modification with 100 kGy) and measure the loss tangent (tan delta), loss modulus E’’, and storage modulus E’ as a temperature function.

The values of storage moduli, loss moduli, and tan delta increase after radiation treatment for all materials, except sorbitol, which corresponds to the change of the values of the moduli at 50% and 100% strain as determined by mechanical tests (Section 3.4.). In the temperatures between −100 and −20 °C, the materials are in a glassy state. In the temperatures between −20 and 0 °C, a significant decrease of storage modulus can be observed. It is linked to a viscoelastic relaxation process associated with the glass transition process. A flexibility plateau can be seen in the temperatures between 0 and 50 °C. In case of PGBSu and PEBSu, the storage modulus values decrease almost to 0, which corresponds to the melting transition shown by DSC analysis. The peak maxima of the loss modulus and loss tangent functions associated with α relaxation correspond well with the glass transition temperature determined by DSC. The peak area of the loss modulus and loss tangent functions is similar for all materials due to the amorphous phase content being comparable for all materials, which is also confirmed by the DSC results. Overall, all of the DMTA results complement well with the results of the DSC analysis.

### 3.5. Mechanical Properties

**Figure 7:**
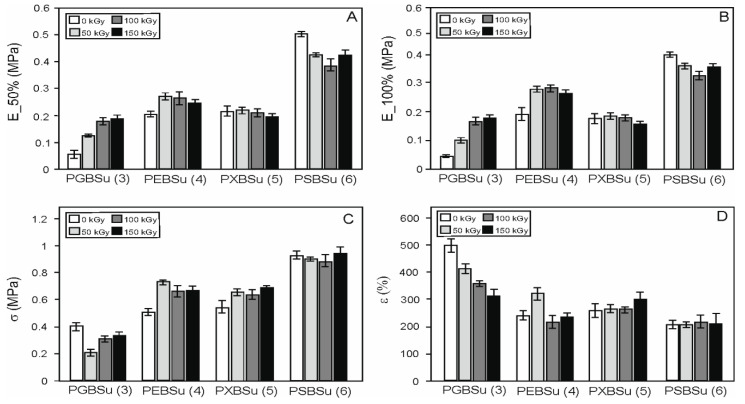
Mechanical properties of PGBSu, PEBSu, PXBSu, and PSBSu before and after irradiation. Tangent modulus at 50% elongation (**A**), tangent modulus at 100% elongation (**B**), stress at break (**C**), and elongation at break (**D**).

In order to examine the radiation treatment effects on the elastomers, mechanical tests (Table 2, Figure 7) were performed. Four materials with different hydroxyl group contents per repeating unit were tested. The materials were as follows: PGBSu (3 hydroxyl groups), PEBSu (4 hydroxyl groups), PXBSu (5 hydroxyl groups), and PSBSu (6 hydroxyl groups). Both non-irradiated materials and materials after modification with 50, 100, and 150 kGy doses were tested. With the increase of hydroxyl group content, an increase of stress at break value and value of moduli at 50% strain can be observed. It is due to higher cross-link density (for PGBSu, PEBSu, PXBSu, and PSBSu).

Radiation modification leads to an increase in stress at break for PEBSu and PXBSu. The values of the moduli at 50% and 100% show an increase for all the irradiation doses for PGBSu, and for 50 and 100 kGy doses for PEBSu. 

The dose of 150 kGy is the most beneficial for PGBSu, the dose of 50 kGy is the most beneficial for PEBSu, and the dose of 100 kGy is the most beneficial for PXBSu. Sorbitol has a very high hydroxyl group content per repeating unit, which leads to the highest cross-link density and highest values of the moduli at 50% and 100% and stress at break. Radiation modification of this material leads only to worsening of those properties. 

### 3.6. Cross-Linking Density

**Figure 8:**
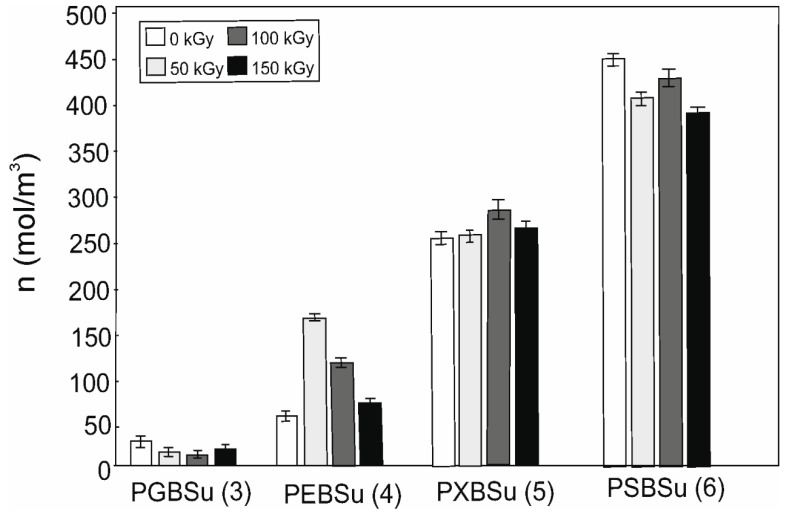
Cross-linking density of non-irradiated and radiation-modified PGBSu, PEBSu, PXBSu, and PSBSu. Hydroxyl group amount is indicated by the numbers in the brackets.

Cross-link density results are shown in Table 2 and Figure 8. Increasing content of the hydroxyl groups is followed by the cross-link density increase (for non-irradiated materials). Radiation treatment leads to decline of the cross-link density for all the doses in case of PGBSu and PSBSu. For PEBSu, the dose of 50 kGy leads to the biggest improvement of cross-link density. The dose of 100 kGy leads to the biggest improvement of cross-link density for PXBSu. 

PEBSu seems to be the best suited for radiation modification because when modified with 50 kGy dose, the cross-link density increases more (as compared with the initial value) than in the case of PXBSu. It is also followed by the most noticeable improvement of mechanical properties exhibited by this material. 

### 3.7. Water Contact Angle

**Figure 9:**
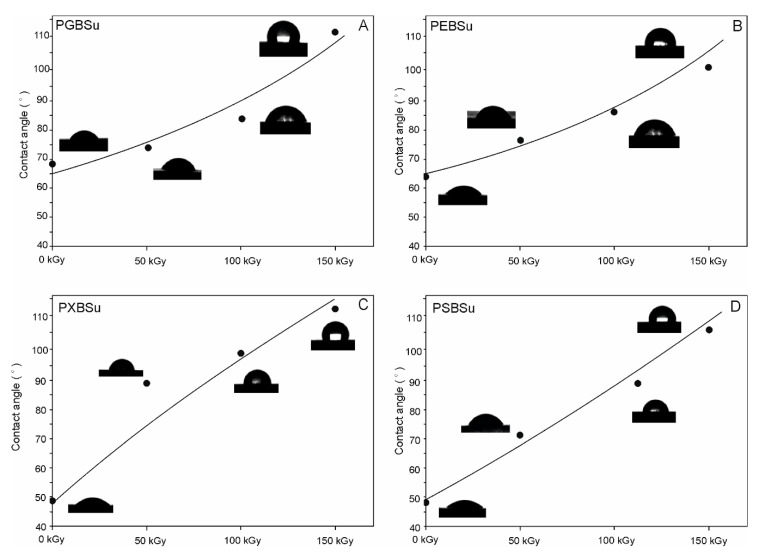
(**A**) PGBSu, (**B**) PEBSu, (**C**) PXBSu, and (**D**) PSBSu water contact angle before and after irradiation.

Results of water contact angle tests are shown in Figure 9 and Table 1. Increasing hydroxyl group content leads to a decrease of water contact angle and increase in hydrophilicity. Applying radiation treatment leads to an increase of hydrophobicity. Higher doses of radiation result in higher hydrophobicity of the affected materials.

## 4. Conclusions

In order to improve the properties of different poly(polyol succinate-co-butylene succinate) elastomers, radiation modification was carried out. Such modification has proven to be simple and effective, materials directly after synthesis were utilized, and no cross-linking agents were necessary. A valuable side effect of this treatment was the sterilization of the materials. 

It has been established that polymers have a semi-crystalline structure with the amorphous phase being dominant. The increasing hydroxyl group content leads to the amorphous phase becoming more prominent, and the cross-link density increasing, which is followed by better mechanical properties.

The influence of various doses of radiation on thermal, mechanical, and chemical properties was analyzed in order to select the dose that is most optimal.

Radiation treatment has proven useful for PEBSu and PXBSu materials, as seen in the increase of cross-link density and improvement of the mechanical properties. PSBSu and PGBSu turned out to be not well suited for such modification, with its properties worsening.

## Figures and Tables

**Table 1:** Selected properties and composition of poly(glycerol succinate-co-butylene succinate) (PGBSu), poly(erythritol succinate-co-butylene succinate) (PEBSu), poly(xylitol succinate-co-butylene succinate) (PXBSu), poly(sorbitol succinate-co-butylene succinate). Data for both non-irradiated and radiation-modified materials is presented.

Material/Dose	Molar Composition (mol)			H ShA	CA	Prepolymer	Molar Composition by ^1^H NMR (mol)		M_w_ (g/mol)	PDI
-	SuA	GL	BG	-		-	GL	BG	-	
PGBSu_0 kGy	2	1	1	38.8 +/− 4.95	68.1 +/− 2.04	PGBSu	0.32	1.00	35,000	1.3
PGBSu_50 kGy				45.8 +/− 4.84	74.1 +/− 1.35					
PGBSu_100 kGy				37.7 +/− 3.34	84.6 +/− 1.15					
PGBSu_150 kGy				38.9 +/− 2.50	111.3 +/− 1.16					
-	SuA	ER	BG				ER	BG	-	
PEBSu_0 kGy	2	1	1	32.7 +/− 2.77	64 +/− 10.15	PEBSu	0.20	1.00	32,000	1.6
PEBSu _50 kGy				43.08 +/− 4.01	76.7 +/− 8.33					
PEBSu_100 kGy				42.8 +/− 5.27	86.4 +/− 9.15					
PEBSu_150 kGy				37.08 +/− 3.45	100.8 +/− 4.20					
-	SuA	XL	BG	-			XL	BG	-	
PXBSu_0 kGy	2	1	1	30.2 +/− 6.77	49.9 +/− 3.05	PXBSu	0.25	1.00	28,000	2.0
PXBSu_50 kGy				41.33 +/− 3.73	88.8 +/− 2.05					
PXBSu_100 kGy				48.25 +/− 2.42	96.8 +/− 2.14					
PXBSu_150 kGy				41.25 +/− 3.08	112.9 +/− 5.12					
-	SuA	SB	BG	-		-	SB	BG	-	
PSBSu_0 kGy	2	1	1	66.8 +/− 2.25	48.7 +/− 2.43	PSBSu	0.22	1.00	24,000	2.3
PSBSu_50 kGy				59.27 +/− 6.38	71.1 +/− 3.24					
PSBSu_100 kGy				71.08 +/− 6.10	89.7 +/− 5.11					
PSBSu _150 kGy				65.83 +/− 1.34	105.4 +/− 4.23					

where SuA: succinic acid, BG: butylene glycol, GL: glycerol, ER: erythritol XL: xylitol, SB: sorbitol, PBS: poly(butylene succinate) segment, PPS: poly(polyol succinate) segment, H: hardness, CA: contact angle.

**Table 2:** Mechanical properties and cross-linking density of poly(glycerol succinate-co-butylene succinate) (PGBSu), poly(erythritol succinate-co-butylene succinate) (PEBSu), poly(xylitol succinate-co-butylene succinate) (PXBSu), poly(sorbitol succinate-co-butylene succinate). Data for both non-irradiated and radiation-modified materials is presented.

Material/Dose	Molar Composition (mol)			E_50% (MPa)	E_100% (MPa)	σ_r_ (MPa)	ε_r_ (%)	*n* (mol/m^3^)
-	SuA	GL	BG	-				
PGBSu_0 kGy	2	1	1	0.07 +/− 0.01	0.06 +/− 0.02	0.40 +/− 0.03	501 +/− 35.09	19.36 +/− 8.66
PGBSu_50 kGy				0.12 +/− 0.1	0.10 +/− 0.01	0.21+/− 0.03	414 +/− 12.01	16.20 +/− 12.32
PGBSu_100 kGy				0.18 +/− 0.03	0.16 +/− 0.03	0.31 +/− 0.05	357 +/− 33.09	12.34 +/− 17.21
PGBSu_150 kGy				0.19 +/− 0.02	0.17 +/− 0.12	0.38 +/− 0.06	312 +/− 32.58	13.20 +/− 21.81
-	SuA	ER	BG	-				
PEBSu_0 kGy	2	1	1	0.204 +/−0.04	0.190+/−0.04	0.509+/−0.12	239 +/− 55.44	66.34 +/−25.22
PEBSu_50 kGy				0.272 +/− 0.11	0.278 +/− 0.11	0.733 +/− 0.19	324 +/− 17.81	165.21 +/− 53.71
PEBSu_100 kGy				0.267 +/− 0.06	0.279 +/− 0.06	0.663 +/− 0.09	215 +/− 68.17	124.93 +/− 33.59
PEBSu_150 kGy				0.248 +/− 0.05	0.261 +/− 0.06	0.669 +/− 0.07	234 +/− 79.12	71.29 +/− 16.88
-	SuA	XL	BG	-				
PXBSu_0 kGy	2	1	1	0.215 +/− 0.06	0.175 +/− 0.05	0.545+/− 0.06	259 +/− 31.64	251.78 +/− 37.32
PXBSu_50 kGy				0.220 +/− 0.05	0.184 +/− 0.05	0.66+/− 0.10	263 +/− 27.75	254.83 +/− 35.24
PXBSu_100 kGy				0.212 +/− 0.03	0.177 +/− 0.03	0.641 +/− 0.06	275 +/− 21.91	287.44 +/− 31.54
PXBSu_150 kGy				0.196 +/− 0.07	0.155 +/− 0.07	0.694 +/− 0.12	301 +/− 66.80	287.51 +/− 36.77
-	SuA	SB	BG	-				
PSBSu_0 kGy	2	1	1	0.503 +/− 0.06	0.396 +/− 0.05	0.93 +/− 0.41	205 +/− 13.03	450.04 +/− 42.19
PSBSu_50 kGy				0.428 +/− 0.11	0.357 +/− 0.10	0.90 +/− 0.09	208 +/− 30.34	414.22 +/− 36.68
PSBSu_100 kGy				0.385 +/− 0.05	0.322 +/− 0.05	0.88 +/− 0.10	215 +/− 13.22	432.16 +/− 47.28
PSBSu _150 kGy				0.426 +/− 0.07	0.355 +/− 0.05	0.95 +/− 0.12	209 +/− 13.70	399.48 +/− 40.82

where: σ_r_: stress at break, ε_r_: elongation at break, E_50%: modulus at 50% elongation, E_100%: modulus at 100% elongation, n: cross-linking density, SuA: succinic acid, BG: butylene glycol, GL: glycerol, ER: erythritol, XL: xylitol, SB: sorbitol.

**Table 3:** First-heating data for differential scanning calorimetry (DSC) analysis of non-irradiated and radiation-modified poly(glycerol succinate-co-butylene succinate) (PGBSu), poly(erythritol succinate-co-butylene succinate) (PEBSu), poly(xylitol succinate-co-butylene succinate) (PXBSu), and poly(sorbitol succinate-co-butylene succinate) (PSBSu).

Material/Dose	*T*_g_	Δ*C*_p_	*T*_m_	Δ*H*_m_
	(°C)	(J/g°C)	(°C)	(J/g)
PGBSu
PGBSu_0 kGy	−25.5	0.749	48.9	2.83
PGBSu_50 kGy	−21.9	0.626	51.3	12.14
PGBSu_100 kGy	−22.1	0.635	51.2	11.79
PGBSu_150 kGy	−22.9	0.719	50.9	12.85
PEBSu
PEBSu_0 kGy	−18.9	0.731	53.8	3.19
PEBSu_50 kGy	−14.7	0.708	55.4	5.24
PEBSu_100 kGy	−14.8	0.701	55.8	4.87
PEBSu_150 kGy	−15.1	0.734	55.1	5.52
PXBSu
PXBSu_0 kGy	−13.7	0.763	-	-
PXBSu_50 kGy	−8.2	0.799	-	-
PXBSu_100 kGy	−7.4	0.759	-	-
PXBSu_150 kGy	−4.5	0.729	-	-
PSBSu
PSBSu_0 kGy	−2.6	0.686	-	-
PSBSu_50 kGy	−0.4	0.764	-	-
PSBSu_100 kGy	1.9	0.729	-	-
PSBSu_150 kGy	−0.3	0.769	-	-

where: *T*_g_: glass transition temperature, ∆*C*_p_: change of the heat capacity, *T*_m_: melting temperature, ∆*H*_m_: melting enthalpy.
